# GNG7 as a tumor-suppressor gene in lung adenocarcinoma: implications for prognosis and immune-based therapies

**DOI:** 10.3389/fonc.2025.1588646

**Published:** 2025-05-27

**Authors:** Kexin Luo, Meihan Liu, Zhongqin Peng, Haiyang Zhao, Guoyi Li, Yuanze Cai, Yumeng Lei, Hongpan Zhang, Yongsheng Zhao

**Affiliations:** ^1^ Affiliated Hospital of North Sichuan Medical College, Nanchong, Sichuan, China; ^2^ Department of Thoracic Surgery, Affiliated Hospital of North Sichuan Medical College, Nanchong, Sichuan, China; ^3^ North Sichuan Medical College Innovation Centre for Science and Technology, Nanchong, China; ^4^ Department of Clinical Medicine, North Sichuan Medical College, Nanchong, China; ^5^ Department of Oncology, Affiliated Hospital of North Sichuan Medical College, Nanchong, China; ^6^ Department of Clinical Medicine, Guizhou Medical University, Guiyang, China

**Keywords:** GNG7, lung adenocarcinoma, immune infiltration, immunotherapy, prognostic model

## Abstract

**Introduction:**

Lung adenocarcinoma (LUAD) is among the most prevalent and lethal forms of cancer worldwide, largely due to the lack of early symptoms and frequent late-stage diagnosis. G protein γ subunit 7 (GNG7) has been implicated in the regulation of cell proliferation, apoptosis, and migration across various cancers. However, its immunological role in LUAD progression remains poorly understood.

**Methods:**

We analyzed The Cancer Genome Atlas (TCGA) database to assess the relationship between GNG7 expression and clinical outcomes in LUAD. Immune cell infiltration and immune-related gene expression were evaluated in association with GNG7 levels. In vitro functional assays, including proliferation, migration, invasion, and apoptosis assays, were performed following GNG7 overexpression. A prognostic model was constructed based on immune-related genes regulated by GNG7 and validated using the GSE31210 and IMvigor210 cohorts.

**Results:**

Low GNG7 expression was associated with enhanced tumor growth and poor prognosis in LUAD patients. GNG7 expression correlated significantly with immune cell infiltration and key immune regulatory markers. In vitro, GNG7 overexpression suppressed LUAD cell proliferation, migration, and invasion, while promoting apoptosis. The developed GNG7-related immune gene prognostic model effectively predicted both patient prognosis and immunotherapy response.

**Discussion:**

Our findings highlight the critical role of GNG7 in LUAD progression and its modulation of the tumor immune microenvironment. GNG7 shows promise as a prognostic biomarker and potential therapeutic target for immune-based LUAD treatment strategies.

## Introduction

Lung cancer remains the most formidable challenge in the oncology realm, Emerging as the leading cause of death connected to cancer worldwide, it stands as the second most commonly diagnosed malignant tumor (1). Among its various subtypes, LUAD notably represents the most prevalent subtype, comprising approximately 40% of all lung cancer cases (2–5). Although advancements have been made in medical treatments such surgery, chemotherapy, radiation therapy, and molecular targeting, the outlook for patients with LUAD remains bleak. The five-year survival rate remains alarmingly low, underscoring an urgent necessity for innovative therapeutic strategies to ameliorate patient outcomes (6, 7).

The emergence of immunotherapy has heralded a promising revolutionary advancements in cancer therapy, particularly with the advent of targeted therapies directed at CTLA-4 and PD-1/PD-L1. These therapies have demonstrated effectiveness in managing a spectrum of solid tumors and have been approved for clinical application (8). However, the heterogeneity of the tumor microenvironment (TME) in LUAD significantly curtails the efficacy of immunotherapy, benefiting only a limited subset of patients (9). This predicament accentuates the critical demand for developing sophisticated prognostic models and discovering novel biomarkers. Such advancements are imperative to accurately forecast therapeutic outcomes and improve prognostication for LUAD patients.

GNG7, a gene encoded within the extensive family of guanine nucleotide-binding proteins gamma on chromosome 19 (10), is implicated in a myriad of transmembrane signaling pathways. Preliminary research has illuminated GNG7’s potential as a suppressor of tumorigenesis, where its overexpression in gastric cancer appears to inhibit cell proliferation and tumorigenicity, primarily through inducing autophagy via the mTOR pathway (11, 12). Furthermore, our *in vitro* experiments have demonstrated that overexpression of GNG7 significantly inhibits cell proliferation, invasion, and migration, while promoting apoptosis, highlighting its potential as a tumor-suppressor gene in LUAD. Despite these intriguing findings, the specific roles and prognostic significance of GNG7 in LUAD, especially concerning its interplay with immune infiltration, remain largely unexplored. Bridging these knowledge gaps presents a unique opportunity to identify novel therapeutic targets and to refine treatment modalities for LUAD.

In response to these challenges, our study endeavors to perform an exhaustive bioinformatics analysis aimed at unraveling the clinical significance and underlying functional mechanisms of GNG7 in LUAD. Through this investigation, we aspire to construct a robust clinical prediction model predicated on our findings. Such a model would not only facilitate a deeper understanding of GNG7’s role within the TME but also potentially guide the formulation of personalized treatment strategies, ultimately enhancing the prognosis and therapeutic responsiveness of LUAD patients.

## Material and methods

### Data collection and pre-processing

Data from The Cancer Genome Atlas (TCGA) database was collected, including clinical information and GNG7 expression patterns, encompassing 33 distinct tumor types. Access to data was facilitated via UCSC Xena (13) (https://xena.ucsc.edu/) and analysis was performed utilizing R software, specifically employing packages suited for genomic data analysis (https://www.R-project.org). The research focuses on extracting somatic mutation information from the TCGA database and building a model, while the Gene Expression Omnibus (GEO) dataset GSE31210 (14) was employed to validate the prognostic capabilities of our model. The efficacy of immunotherapy drugs is verified by the IMvigor210 cohort of atezolizumab in the treatment of urothelial cancer (15).

### The correlation between GNG7 and pan-cancer

We investigated GNG7 expression differences and their correlation with clinical factors (gender, age, tumor stage) using R software. Our analysis included univariate Cox regression with R’s survival package to assess GNG7’s prognostic significance in 33 cancers, focusing on overall survival (OS). A hazard ratio (HR) greater than 1 suggested higher GNG7 expression increases mortality risk. For defining high/low expression of GNG7, we used the median expression value across all tumor types as a cutoff (threshold: 0.5). We also examined GNG7’s relationship with clinical stage, grade, and TNM stage, using a p-value < 0.05 as the significance threshold.

### Analysis of immune cell infiltration and immune characteristics

We employed the CIBERSORT (16) algorithm to quantify the abundances of 22 immune cell types and analyze their association with GNG7 expression. We used the ESTIMATE algorithm to determine the immunological score, which assesses the level of immune cell infiltration, comparing samples with high versus low expression of the gene (cutoff: median expression value of GNG7). Spearman’s correlation analysis was used to examine the relationship between gene expression and immunological scores. Additionally, the MCPcounter (17) method was used to estimate cellular scores, offering insights into the specific cell types present within the tumor microenvironment. The significance threshold for immune infiltration analysis was set to a p-value < 0.05.

### Genomic screening and gene set enrichment analysis

Utilizing Spearman’s correlation, we conducted GO enrichment analysis on genes associated with GNG7 and immune infiltration, selecting those significantly correlated with GNG7 expression and immune scores. Only correlation coefficients > 0.3 or < -0.3 were significant. We performed KEGG enrichment analysis on standard genes using the clusterProfiler R software package, including cellular components (CC), molecular functions (MF), and biological processes (BP). We prioritized the top 10 most significant terms from each category for visualization. We used the Gene Set Variation Analysis (GSVA) approach to calculate pathway scores for TCGA sample cohorts in pathway analysis. Differences in pathway activity between the GNG7 high and low expression groups were evaluated using the Wilcoxon test. Moreover, single-sample Gene Set Enrichment Analysis (ssGSEA) was utilized to explore correlations among these significantly differing pathways.

### Risk model construction

Genes with significant prognostic value were selected through univariate Cox and Lasso regression analyses. For the definition of high/low-risk groups, we computed risk scores for each subject via multivariate Cox regression analysis, categorizing them into high or low-risk groups based on their z-scores (cutoff: median value of the risk scores across all subjects). The performance of the model was validated using an independent dataset from GEO. For validation, an independent dataset from the GEO was utilized. Comprehensive assessments including univariate and multivariate Cox analyses, nomograms, and Decision Curve Analysis (DCA).

### Immunotherapy response and chemotherapy analysis

In this investigation, we analyzed an independent cohort undergoing immunotherapy, categorizing treatment outcomes into four efficacy categories: complete response (CR), partial response (PR), progressive disease (PD), and stable disease (SD). Non-responders were identified as those falling into the SD or PD categories, whereas responders were classified based on achieving either CR or PR. To evaluate the variance in GNG7 expression between responder and non-responder groups, we employed the Wilcoxon rank-sum test (cutoff: median GNG7 expression level). A p-value < 0.05 was considered statistically significant.

### Cell culture

The BEAS-2B cell line was purchased from Baidi Biotechnology Co., Ltd. (Zhejiang, China). The A549 and H1299 cell lines were obtained from Wuhan Pricella Biotechnology Co., Ltd. (Wuhan, China). All cell lines were cultured in DMEM medium supplemented with 10% fetal bovine serum (FBS) and 1% penicillin-streptomycin (pen-strep) at 37°C in a 5% CO2 incubator, according to the supplier’s instructions.

### RNA extraction and reverse transcription quantitative PCR (RT-qPCR)

Total RNA was extracted from the samples using TRIzol reagent, followed by homogenization, chloroform addition, and centrifugation. The aqueous phase containing RNA was collected and precipitated with isopropanol, then washed with 75% ethanol to remove impurities. RNA concentration and quality were assessed using a spectrophotometer. Reverse transcription was performed using a commercial kit according to the manufacturer’s protocol. The cDNA was used for qPCR analysis on a Bio-Rad CFX96 Real-Time PCR System, with the following primers: GNG7 forward: ATGTCAGCCACTAACAACATAGC, reverse: AGACCTTGATGCGCTCAATCC; GAPDH forward: CTTTGGTATCGTGGAAGGACTC, reverse: GTAGAGGCAGGGATGATGTTCT. PCR conditions were as follows: initial denaturation at 95°C for 2 minutes, followed by 40 cycles of 95°C for 15 seconds and 60°C for 30 seconds. Relative expression levels of GNG7 were calculated using the ΔΔCt method. For GNG7 overexpression analysis, plasmids obtained from Genepharma were amplified with the same PCR conditions.

### Western blot analysis

Protein extraction was performed using RIPA lysis buffer (Epizyme Biomedical Technology), followed by ultrasonic disruption of the cells. After lysis, samples were centrifuged at 12,000 × g for 15 minutes at 4°C. Protein concentration was determined using the BCA assay. For SDS-PAGE, 20-50 μg of protein was denatured in SDS sample buffer at 100°C for 10 minutes and separated on a 10-15% polyacrylamide gel. The proteins were then transferred to a pre-activated PVDF membrane (Millipore) at 250 mA for 1 hour at 4°C. After transfer, the membrane was blocked with a rapid blocking solution for 20–30 minutes and incubated overnight at 4°C with primary antibody (Affinity). The membrane was then washed with TBST and incubated with HRP-conjugated secondary antibody (1:5000) for 1 hour at room temperature. Following washes, protein bands were detected using ECL reagents (Epizyme Biomedical Technology) and visualized with a chemiluminescence imaging system. Band intensity was quantified using ImageJ software, and relative protein expression was calculated as the ratio of the target protein to the loading control.

### Cell viability assay

Cell viability was assessed using the Cell Counting Kit-8 (CCK-8) assay (18). A total of 6000–7000 cells were seeded into each well of a 96-well plate, with a final volume of 100 µL per well, and cultured to the desired density. Afterward, 10 µL of CCK-8 solution was added to each well and mixed gently. The cells were incubated at 37°C with 5% CO_2_ for 1 hour. The absorbance at 450 nm was measured using a microplate reader, which correlates directly with the number of viable cells. Each experiment was repeated three times to ensure reproducibility. Statistical analysis was performed using a Student’s t-test for comparisons between two groups.

### Transwell migration and invasion assays

Cell migration and invasion were assessed using Transwell chambers with an 8 µm pore membrane (Corning, USA). For the migration assay, 5 × 10^4 cells were seeded in the upper chamber with serum-free medium, and the lower chamber contained 10% FBS as a chemoattractant. After 24 hours of incubation at 37°C with 5% CO_2_, non-migrated cells on the upper membrane were removed, and migrated cells on the underside of the membrane were fixed with 4% paraformaldehyde, stained with 0.1% crystal violet, and counted under a microscope in five random fields per membrane. For the invasion assay, the procedure was similar, except the membrane was pre-coated with Matrigel (Beyotime, China). Cells were seeded in serum-free medium and incubated for 48 hours. Cells that invaded through the Matrigel and adhered to the lower membrane were fixed, stained, and counted following the same method as the migration assay. All assays were performed in triplicate, statistical analysis was performed using a Student’s t-test for comparisons between two groups.

### Cell scratch assay

A total of 5×10^5 cells were seeded in a 6-well plate and cultured to confluence. After 24 hours, a linear scratch was made in the monolayer using a sterile 200 µL pipette tip. The medium was then replaced with 1% serum-containing medium to minimize cell proliferation and focus on migration. Images of the wound area were captured immediately after scratching (t = 0) and at subsequent time points (24 and 48 hours) using a phase-contrast microscope. Cell migration was quantified by measuring the gap distance in the images and calculating the percentage of wound closure relative to the initial wound area. The experiment was conducted in triplicate, and data were analyzed using a Student’s t-test to evaluate the effects of time and conditions on cell migration.

### Flow cytometry analysis of apoptosis

Apoptosis was evaluated using Annexin V-FITC/PI staining followed by flow cytometry. Cells were collected, washed with cold PBS, and resuspended in binding buffer (Key GEN Biotech, Annexin V-FITC/PI Kit). To detect early apoptosis, 5 µL of Annexin V-FITC was added to 1×10^6 cells/mL and incubated at room temperature for 10 minutes. Afterward, 10 µL of propidium iodide (PI) was added, and cells were incubated for an additional 5 minutes to stain late apoptotic and necrotic cells. A total of 20,000 events were analyzed using an Agilent flow cytometer, and the data were processed with FlowJo software. Each experiment was repeated three times, and statistical analysis was performed using the paired Student’s t-test.

### Statistical analysis

Statistical analysis was performed using R software. Data are presented as the mean ± SD for continuous variables and as percentages for categorical variables. For comparisons between two groups, Student’s t-test (for normally distributed data) or the Wilcoxon rank-sum test (for non-normally distributed data) was used. For categorical data, the chi-square test was applied. Pearson or Spearman correlation was used to assess the relationships between continuous variables. A p-value < 0.05 was considered statistically significant. All experiments were performed at least in triplicate to ensure statistical robustness.

## Results

### Differential expression of GNG7 in cancer and its prognostic value across cancer subtypes

**Figure 1:**
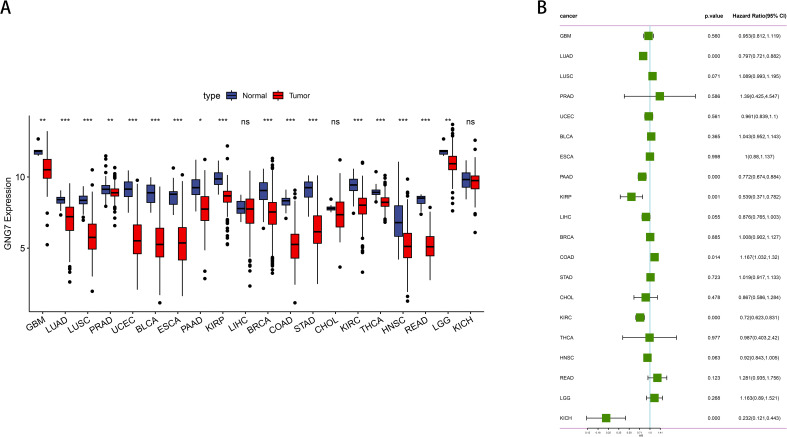
Expression and prognostic analysis of GNG7 in pan-cancer. **(A)** Differential expression analysis of GNG7 in tumor versus normal tissues across various cancer types based on TCGA data. GNG7 expression was significantly downregulated in multiple tumors including GBM, LUAD, LUSC, KIRC, BLCA, ESCA, PAAD, BRCA, KIRP, PRAD, COAD, STAD, THCA, UCEC, HNSC, READ, and LGG (*P*<0.05). **(B)** Univariate Cox regression analysis of GNG7 expression and overall survival. High GNG7 expression is associated with better prognosis in LUAD, PAAD, KIRP, KIRC, and KICH, but worse prognosis in COAD (*p*<0.05). *p<0.05, **p<0.01, ***p<0.001, ns = not significant.

We employed the TCGA database to analyze the expression levels of GNG7 across various cancer types as well as normal tissue samples. Our analysis revealed that, compared to normal tissues of various cancer types, GNG7 expression is significantly reduced in tumor samples, including Glioblastoma multiforme (GBM), LUAD, lung squamous cell carcinoma (LUSC), kidney renal clear cell carcinoma (KIRC), bladder urothelial carcinoma (BLCA), esophageal carcinoma (ESCA), pancreatic adenocarcinoma (PAAD), breast invasive carcinoma (BRCA), kidney renal papillary cell carcinoma (KIRP), prostate adenocarcinoma (PRAD), colon adenocarcinoma (COAD), stomach adenocarcinoma (STAD), thyroid carcinoma (THCA), uterine corpus endometrial carcinoma (UCEC), head and neck squamous cell carcinoma (HNSC), rectum adenocarcinoma (READ), and lower grade glioma (LGG). (*P*<0.05, **Figure 1A**). Additionally, we conducted COX survival analyses based on GNG7 expression, indicating that a higher expression level is associated with improved survival rates in LUAD, PAAD, KIRP, KIRC, Kidney Chromophobe (KICH). Conversely, high GNG7 expression correlates with a poorer prognosis in colorectal adenocarcinoma (COAD) (*P*<0.05, **Figure 1B**).

### Impact of GNG7 expression on clinical outcomes in LUAD

**Figure 2:**
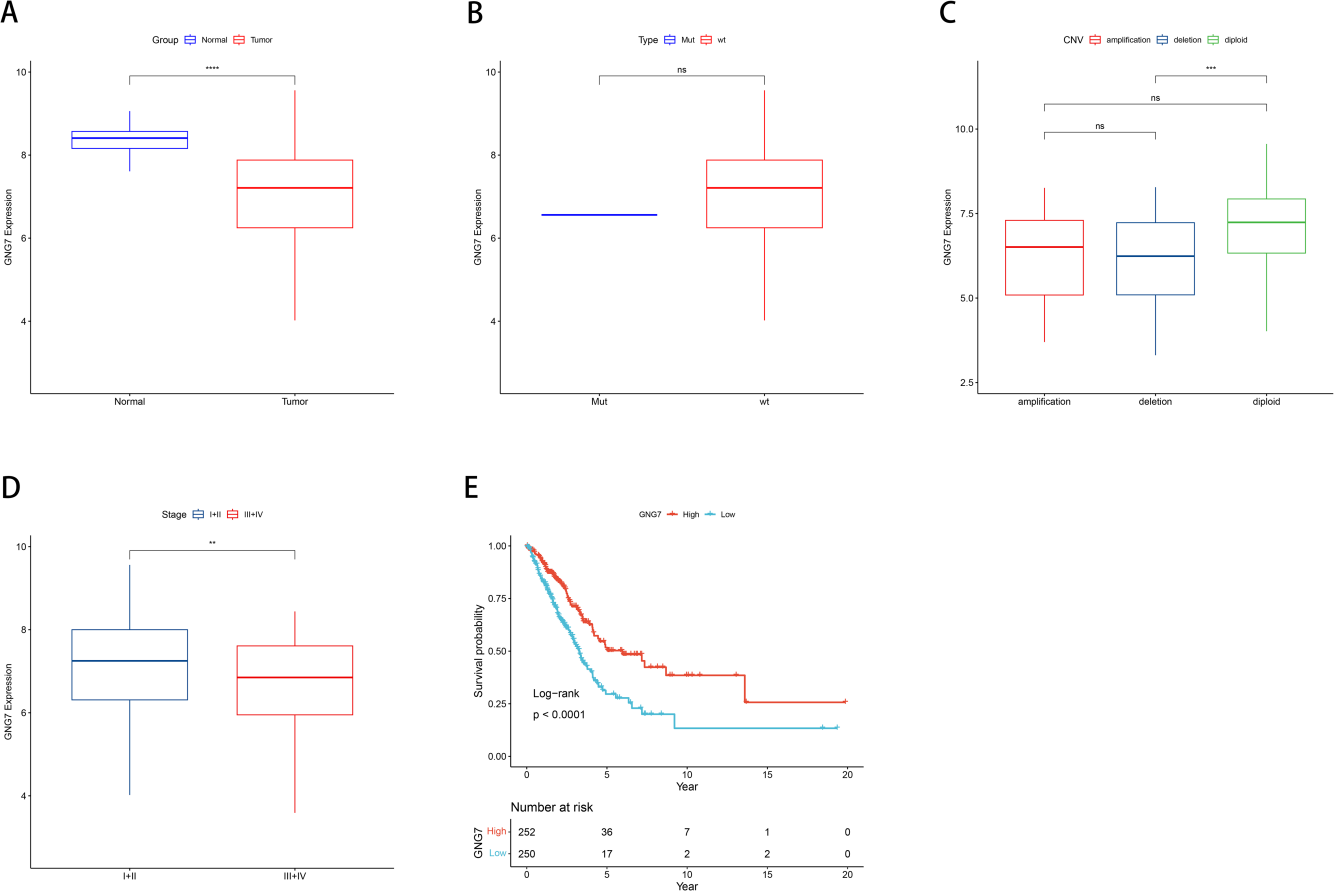
Correlation between GNG7 expression and clinical features in LUAD. **(A)** GNG7 expression is significantly lower in tumor tissues compared to normal tissues (*P*<0.0001). **(B)** No significant difference in GNG7 expression between gene mutation and wild-type groups. **(C)** GNG7 expression is reduced in samples with copy number deletions compared to diploid samples (*P*<0.001). **(D)** Lower GNG7 expression is observed in advanced cancer stages (III–IV) compared to early stages (I–II) (*P*<0.01). **(E)** Kaplan–Meier survival curves show that low GNG7 expression is associated with poorer overall survival (*P*<0.0001). CNV, Copy Number Variation; K-M, Kaplan–Meier. **p<0.01, ***p< 0.001, ****p< 0.0001, ns = not significant.

Leveraging data from the TCGA databases, we concentrated on the significance of GNG7 expression in cancer, analyzing its expression in normal versus tumor tissues, association with gene mutations and CNVs (Copy Number Variations), and correlation with cancer staging and patient survival. The findings revealed that GNG7 expression levels in tumor tissues were notably lower compared to those in normal tissues (*P*<0.0001, **Figure 2A**). Comparisons between the mutant and wild-type groups did not reveal significant differences in GNG7 expression (**Figure 2B**). Analysis of GNG7 expression across different CNV categories indicated that diploid samples exhibited significantly higher expression compared to samples with deletions (*P*<0.001, **Figure 2C**). When contrasting stages I-II and stages III-IV cancer stages, a notable discrepancy in GNG7 expression was noted, with reduced expression linked to advanced cancer stages (*P*<0.01, **Figure 2D**). Additionally, K-M curves illustrated that patients exhibiting individuals with low GNG7 expression had considerably lower survival rates compared to those with high expression levels. (*P*<0.0001, **Figure 2E**).

### Correlation of GNG7 expression with immune infiltration and the tumor microenvironment

**Figure 3:**
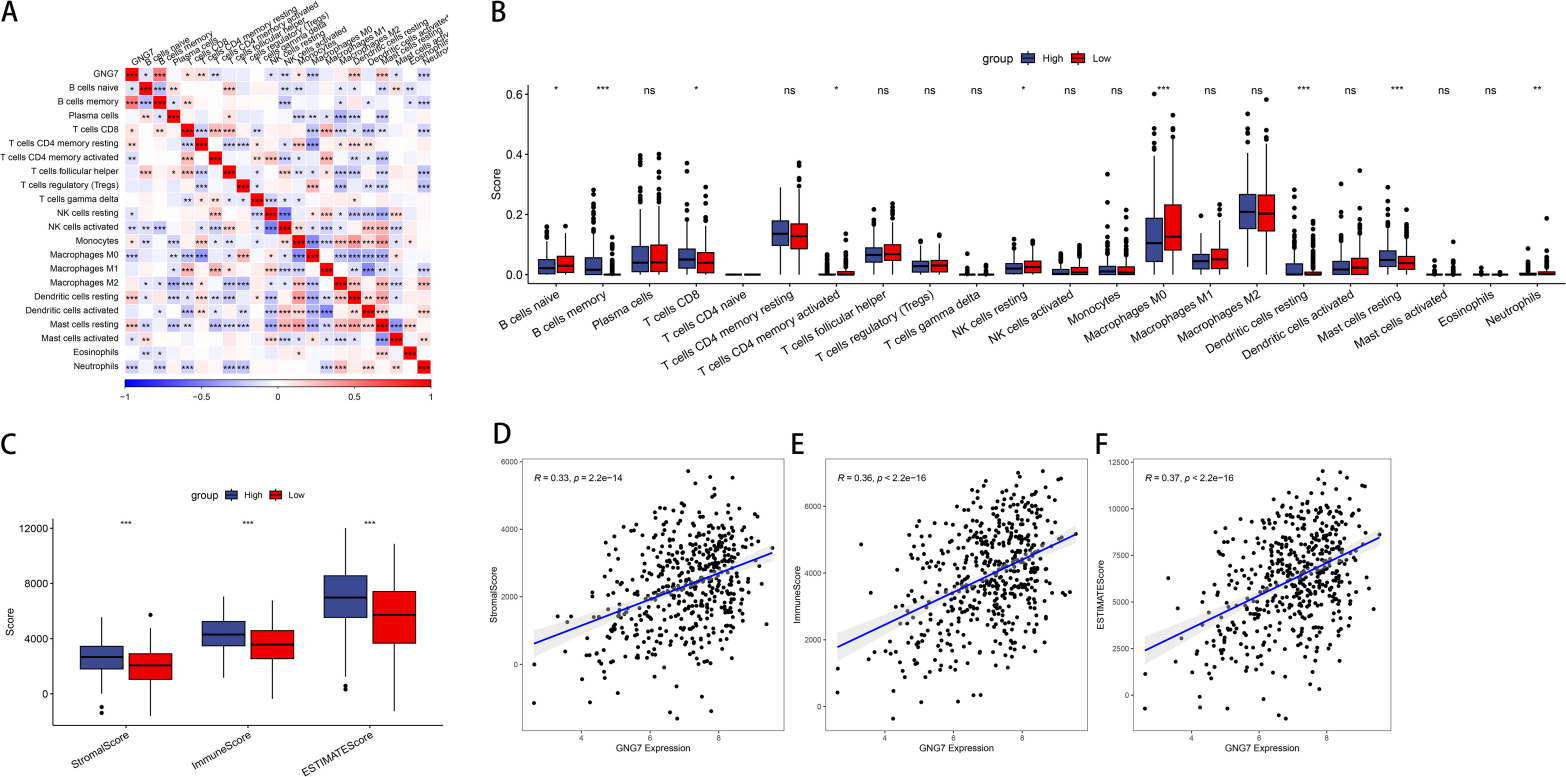
Association of GNG7 expression with immune cell infiltration in LUAD. **(A, B)** GNG7 expression is positively correlated with CD8+ T cells, resting dendritic cells, memory B cells, and resting mast cells, but negatively correlated with immature B cells, M0 macrophages, neutrophils, activated CD4+ memory T cells, and resting NK cells (*P*<0.05). **(C)** Stromal, immune, and ESTIMATE scores differ significantly between GNG7 high- and low-expression groups (*P*<0.001). **(D–F)** GNG7 expression positively correlates with stromal score, immune score, and ESTIMATE score. *p<0.05, **p<0.01, ***p<0.001, ns = not significant.

We calculated the immune scores of 22 types of immune cells, and used the median to divide high and low expression groups to compare the differences in immune cell scores between high and low GNG7 expression groups. We observed that GNG7 expression significantly correlates with the infiltration levels of various immune cells in the tumor microenvironment. Notably, higher GNG7 expression positively correlates with the infiltration levels of CD8+ T cells, resting dendritic cells, memory B cells, and resting mast cells, while negatively correlates with the immature B cells, M0 macrophages, neutrophils, CD4 memory activated T cells, and resting NK cells (*P*<0.05, **Figures 3A, B**). Moreover, we analyzed the relationship between GNG7 high and low expression groups and stromal score, immune score, and ESTIMATE score, and found significant differences (*P*<0.001, **Figure 3C**). The expression of GNG7 was significantly positively correlated with stromal score, immune score and ESTIMATE score (**Figures 3D-F**).

### Related analysis of GNG7 expression pathway

**Figure 4:**
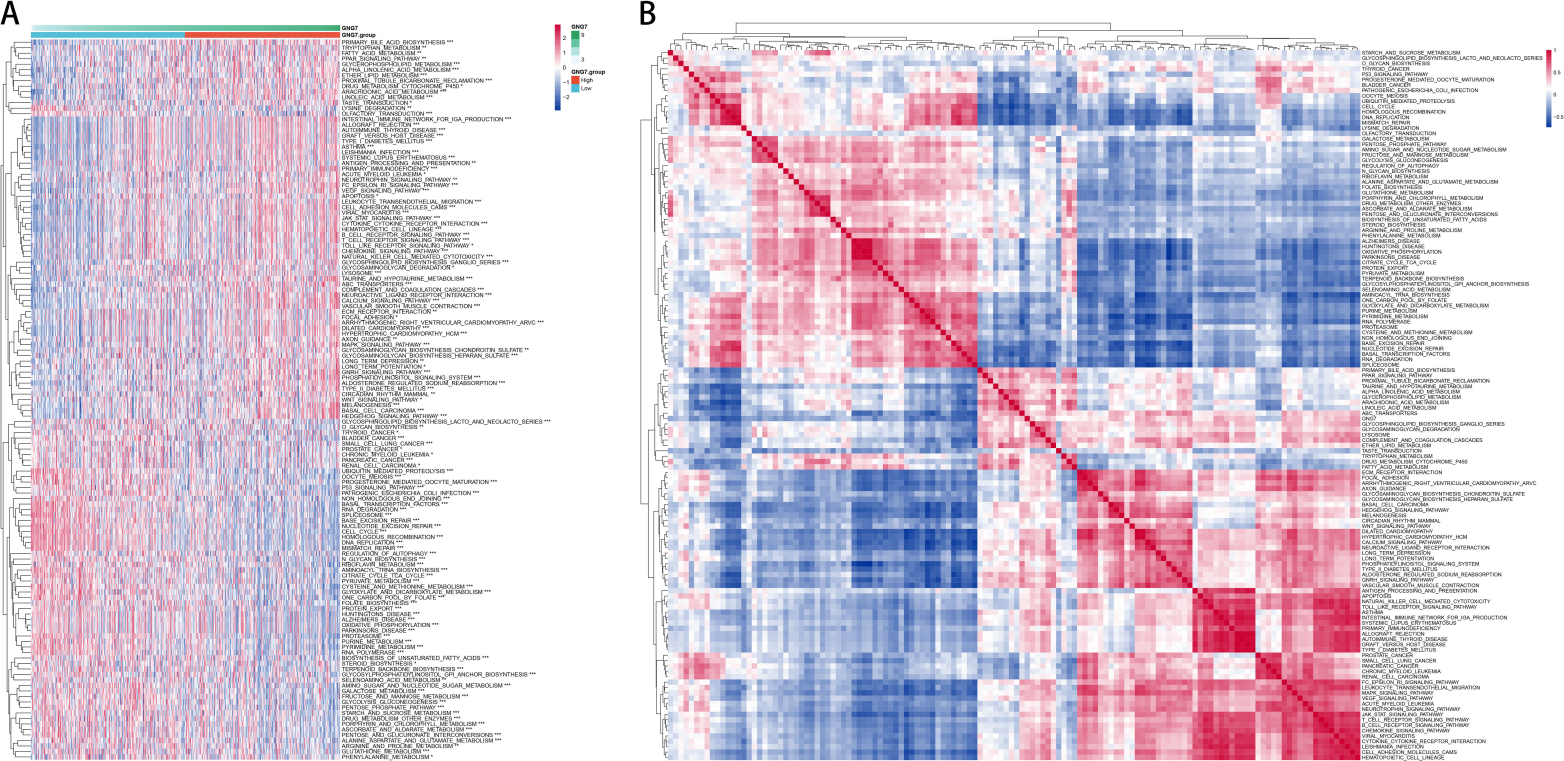
Functional enrichment of GNG7-related pathways in cancer. **(A)** GSVA heatmap showing distinct pathway enrichment profiles in GNG7 high- and low-expression groups. **(B)** ssGSEA-based pathway correlation analysis reveals functional differences between groups. GSVA, Gene Set Variation Analysis; ssGSEA, single-sample Gene Set Enrichment Analysis.

To further explore the correlation between GNG7 expression and biological functions, we performed gene set variation analysis (GSVA) on the TCGA expression profiles using R software, generating single-sample enrichment scores (ssGSEA) for each pathway across individual samples. The heatmap in **Figure 4A** illustrates the differential activation of pathways between the high and low GNG7 expression groups. Notably, immune-related pathways such as antigen processing and presentation and natural killer cell-mediated cytotoxicity were enriched in the high GNG7 expression group, while pathways associated with tumor progression and metabolism, including cell cycle and glycolysis/gluconeogenesis, were upregulated in the low expression group.

Additionally, **Figure 4B** shows the correlation patterns among significantly enriched pathways, revealing functionally clustered modules within each GNG7 subgroup. These findings suggest that GNG7 expression is closely linked to distinct biological processes, potentially influencing both immune activity and tumor behavior in LUAD.

### Comprehensive analysis of GNG7 correlation with cancer immune signature genes and functional enrichment

**Figure 5:**
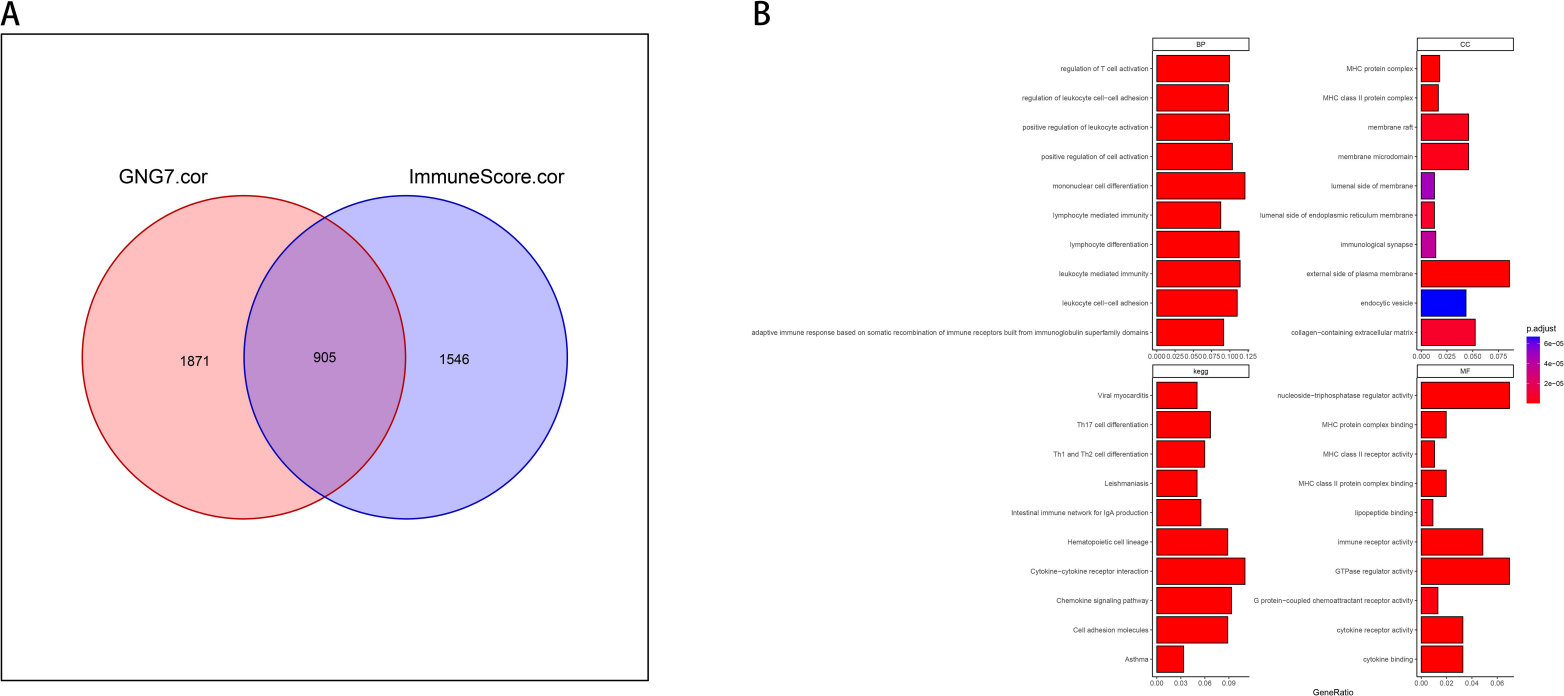
Immune-related functional enrichment of GNG7-correlated genes. **(A)** Venn diagram of 905 intersecting genes correlated with both GNG7 expression and immune scores (|*r*| > 0.3). **(B)** GO and KEGG enrichment of overlapping genes highlights pathways associated with immune function, including T cell activation, leukocyte adhesion, and cytokine receptor activity. BP, biological process; CC, cellular component; MF, molecular function.

In our study, we took an integrative approach to elucidate the relationship between GNG7 expression, immune system interactions, and cancer biological functions. Initially, we used GO enrichment analysis to select genes with correlation coefficients greater than 0.3 or less than -0.3, creating Venn diagrams to describe the overlap between genes associated with GNG7 expression and genes associated with immune scores in the TME. We took the intersection of all genes with GNG7 correlation >0.3 or <-0.3 and immune score correlation >0.3 or <-0.3, and obtained 905 genes (**Figure 5A**). Subsequently, the 905 shared genes were enriched and analyzed through the clusterProfiler package, and the top 10 most significant entries were selected for visualization, providing an overview of biological processes (BP), cellular components (CC), and molecular functions (MF) and kegg’s insights. We observed significant enrichment in BP, such as T cell activation and leukocyte cell adhesion regulation, CC containing MHC protein complexes, MF related to cytokine receptor activity, and pathways enriched in KEGG are all related to immunity (**Figure 5B**).

### Build and validate the LUAD risk model

**Figure 6:**
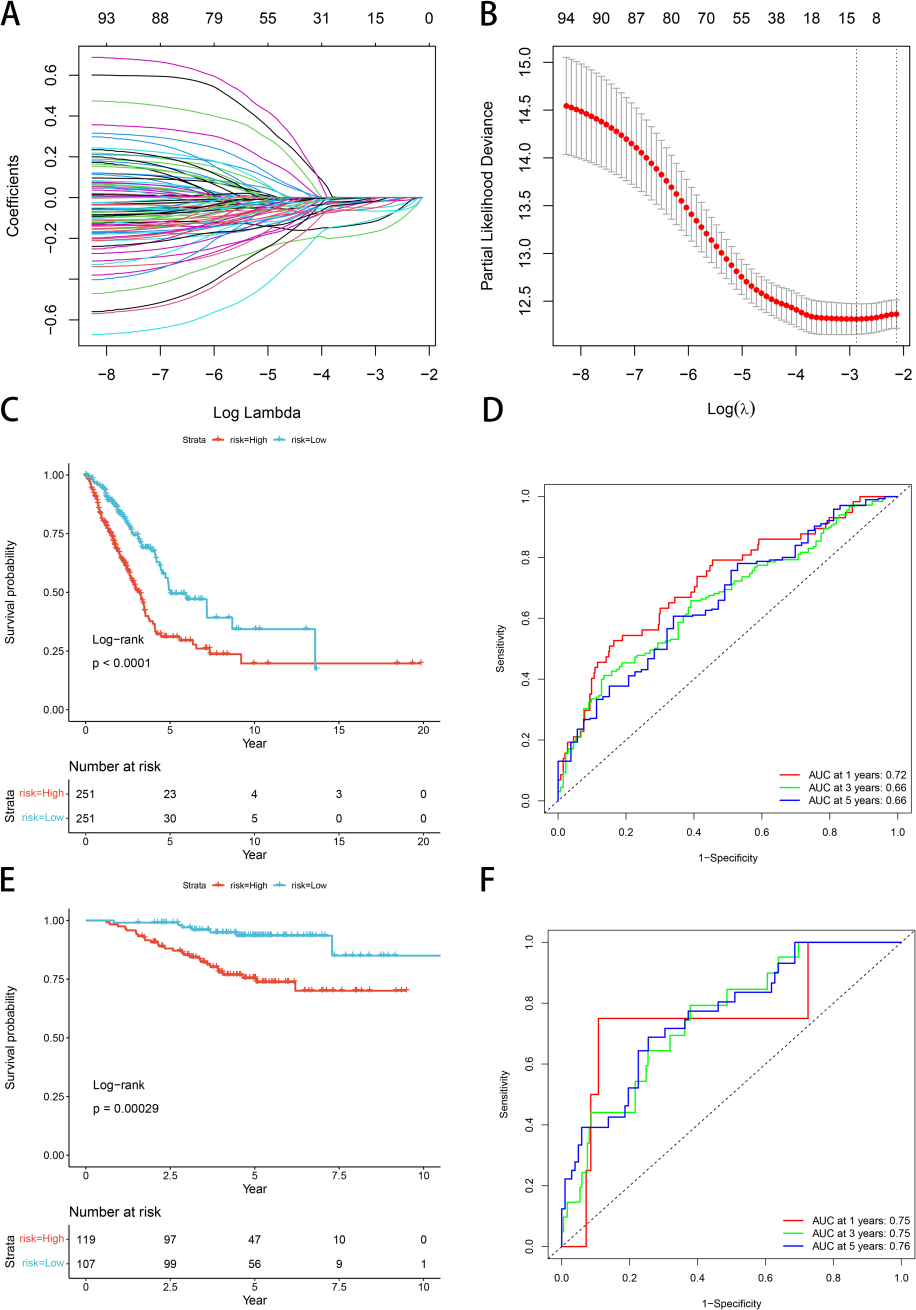
Construction and validation of a GNG7-based LUAD prognostic risk model. **(A, B)** LASSO Cox regression identifies 11 prognostic genes from TCGA LUAD cohort. **(C)** Kaplan–Meier curve shows significantly better OS in the low-risk group (*P*<0.0001). **(D)** ROC curves for 1-, 3-, and 5-year OS show strong predictive performance. **(E, F)** GSE31210 validation confirms model reliability (KM *P*=0.00029; AUCs: 1-year=0.75, 3-year=0.75, 5-year=0.76). ROC, receiver operating characteristic; AUC, area under the curve.

Univariate Cox proportional hazards regression models were performed using expression profiling data from TCGA and associated gene and survival data. Subsequently, 93 genes were identified as having prognostic value, and lasso regression was used to further compress these 93 genes and reduce the number of genes in the risk model. (**Figure 6A**). Finally, we obtained 11 genes: BTN2A2, C17orf44, CBFA2T3, CLEC17A, DENND1C, GGA2, SETDB2, TESK2, XCR1, ZNF136, and ZNF25 (**Figure 6B**). We calculated risk scores by multifactor cox analysis and performed z-score on risk scores. The K-M curve illustrated that patients classified as low-risk exhibited a more favorable prognosis compared to those classified as high-risk (*P*<0.0001, **Figure 6C**). ROC analysis demonstrated that the model in the TCGA dataset had significant predictive value for LUAD patients (1-year AUC=0.72, 3-year AUC=0.66, and 5-year AUC=0.66) (**Figure 6D**). We used GSE31210 to validate the risk model proposed in this work. The risk score of each sample was computed based on its expression level, and the distribution of risk scores across the samples was plotted. The results obtained by the K-M curve are consistent with our model (*P*=0.00029, **Figure 6E**). High-risk patients had poor prognosis, as ROC analysis showed (1-year AUC=0.75, 3-year AUC=0.75, 5-year AUC=0.76) (**Figure 6F**).

### Association of LUAD clinicopathological features with risk models

**Figure 7:**
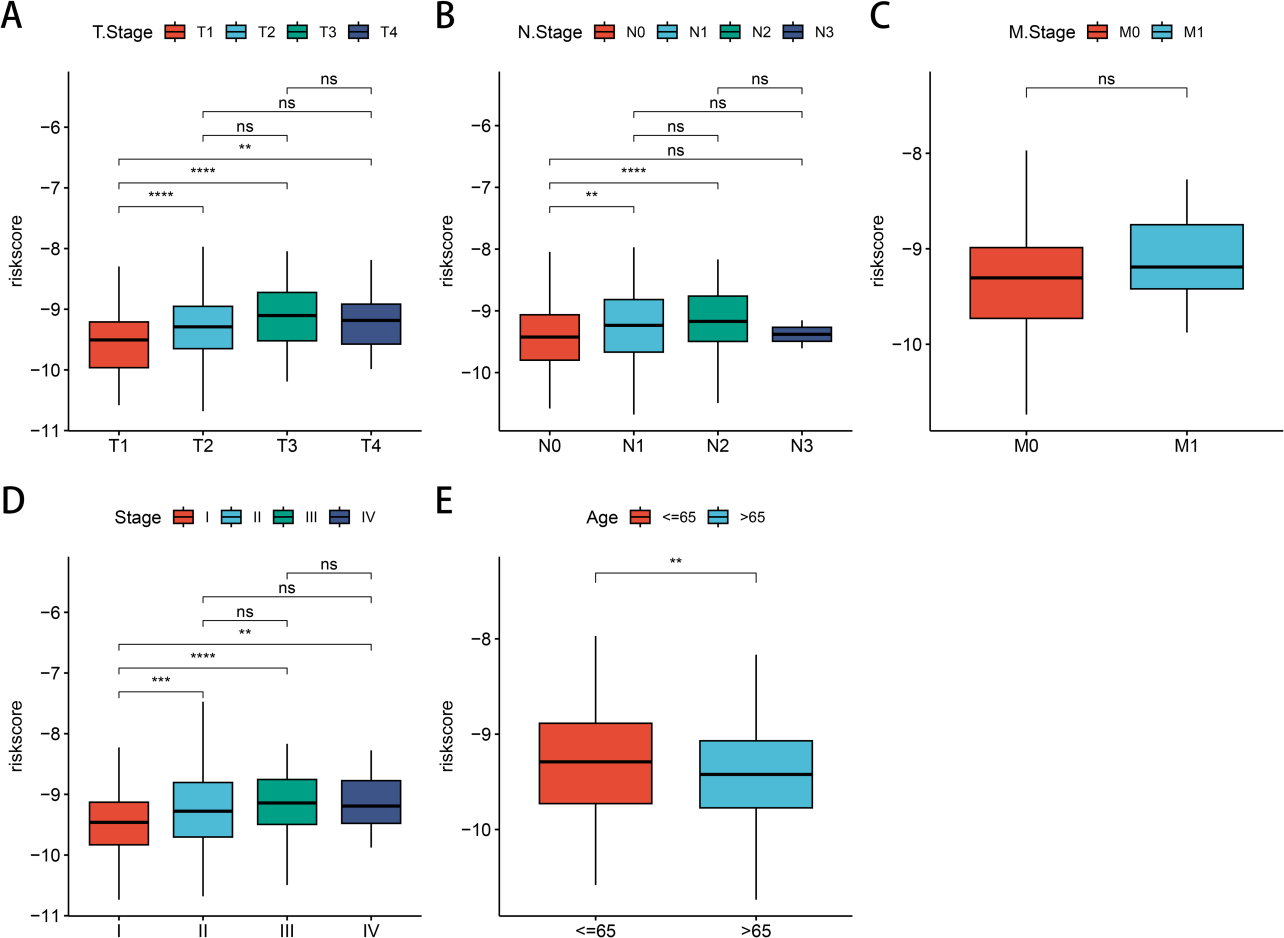
Association between LUAD clinical characteristics and GNG7 risk score. **(A–D)** Risk score increases with tumor (T), node (N), metastasis (M), and clinical stage (*P*<0.05). **(E)** Patients over 65 years show lower risk scores. **p<0.01, ***p<0.001, ****p< 0.0001, ns = not significant.

Subsequently, we observed the relationship between clinical phenotypes and risk models and found that the patients’ (T) stage (**Figure 7A**), (N) stage (**Figure 7B**), (M) stage (**Figure 7C**), and clinical stage (**Figure 7D**) Median risk scores all increased with tumor stage progression, emphasizing the prognostic relevance of GNG7 risk score and cancer progression. However, the opposite phenomenon occurred for age scores, with LUAD patients over 65 years of age having reduced risk scores (**Figure 7E**).

### Correlation analysis between LUAD risk score expression and immune cell infiltration

**Figure 8:**
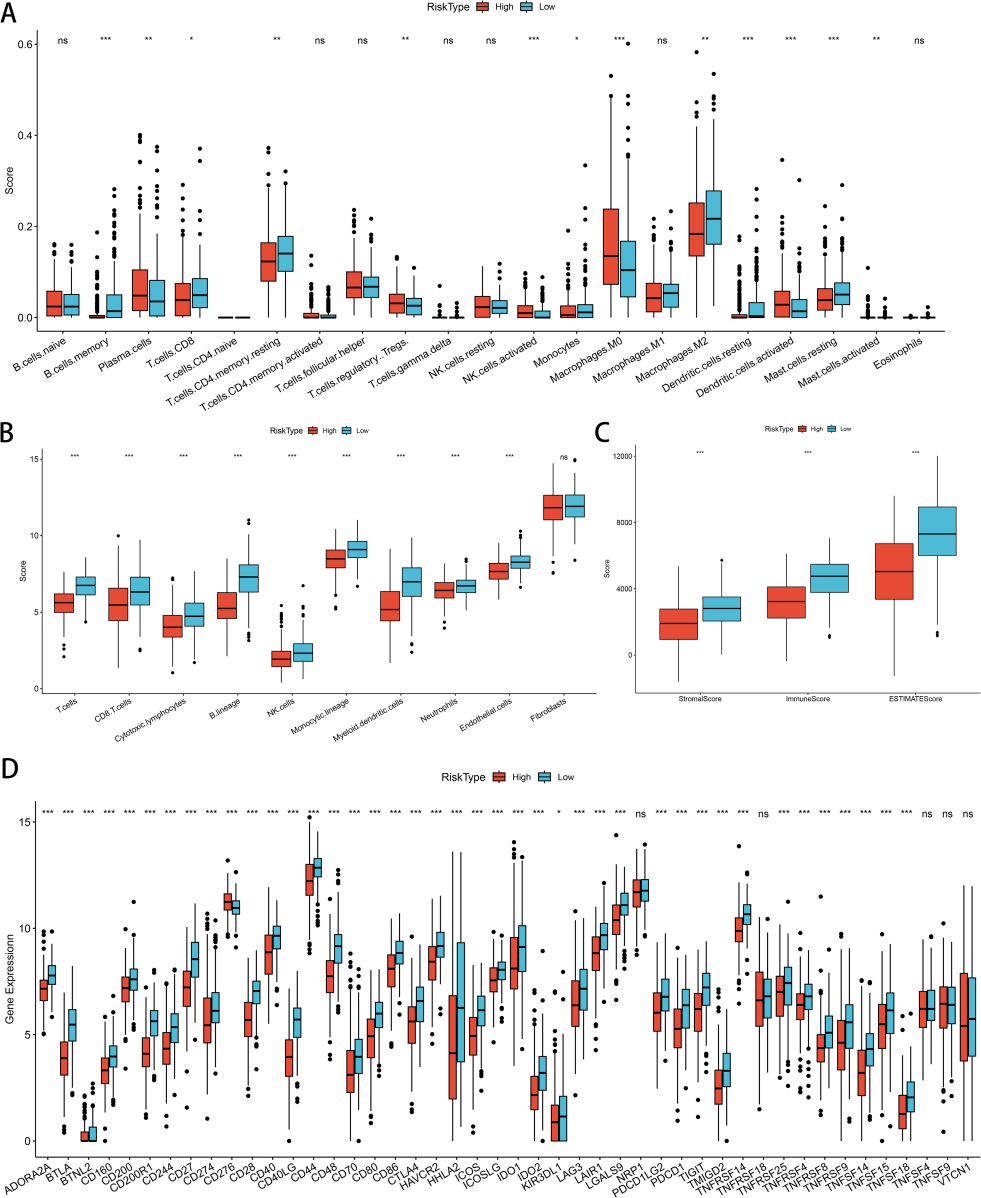
Correlation of GNG7 risk score with immune cell infiltration and immune characteristics in LUAD. **(A)** Low-risk group shows higher infiltration of memory B cells, CD4+/CD8+ T cells, monocytes, M2 macrophages, and resting dendritic/mast cells (*P*<0.05). **(B)** Immune signature scores are significantly elevated in the low-risk group (*P*<0.001). **(C)** Stromal, immune, and ESTIMATE scores are higher in the low-risk group (*P*<0.001). **(D)** Immune checkpoint genes such as CTLA4, IDO1, and BTLA are more highly expressed in the low-risk group (*P*<0.001). *p<0.05, **p<0.01, ***p<0.001, ns = not significant.

Initially, we compared immune cell populations between different risk groups for GNG7 to examine differences in immune cell infiltration levels. The analysis revealed significant disparities in the infiltration levels of various immune cells. Specifically, Memory B cells, CD4+ T cells, CD8+ T cells, monocytes, M2 macrophages, resting dendritic cells, and resting mast cells were more abundant in the low-risk group. Conversely, the high-risk group demonstrated higher infiltration of plasma cells, Tregs, activated NK cells, activated dendritic cells, M0 macrophages and activated mast cells (*P*<0.05, **Figure 8A**). Subsequently, we conducted a comprehensive assessment of immune feature scores predicted for cells between the two groups. Remarkably, the low-risk group exhibited significantly higher scores in T cells, CD8+ T cells, cytotoxic lymphocytes, B lineage cells, NK cells, monocytes, myeloid dendritic cells, neutrophils and endothelial cells, indicating notable differences in immune characteristics *(P*<0.001, **Figure 8B**). Moreover, the matrix score, immune score, and ESTIMATE score of the GNG7 low-risk group were significantly higher compared to those of the high-risk group, further underscoring the substantial differences in the immune environment between the two groups (*P*<0.001, **Figure 8C**). Analysis of immune checkpoint-related gene expression in the risk-differentiated expression group showed considerably higher expression of most immune checkpoint-related genes, including CTLA4, IDO1, and BTLA, in the low-risk group (*P*<0.001, **Figure 8D**).

### Immunotherapy dataset validation

**Figure 9:**
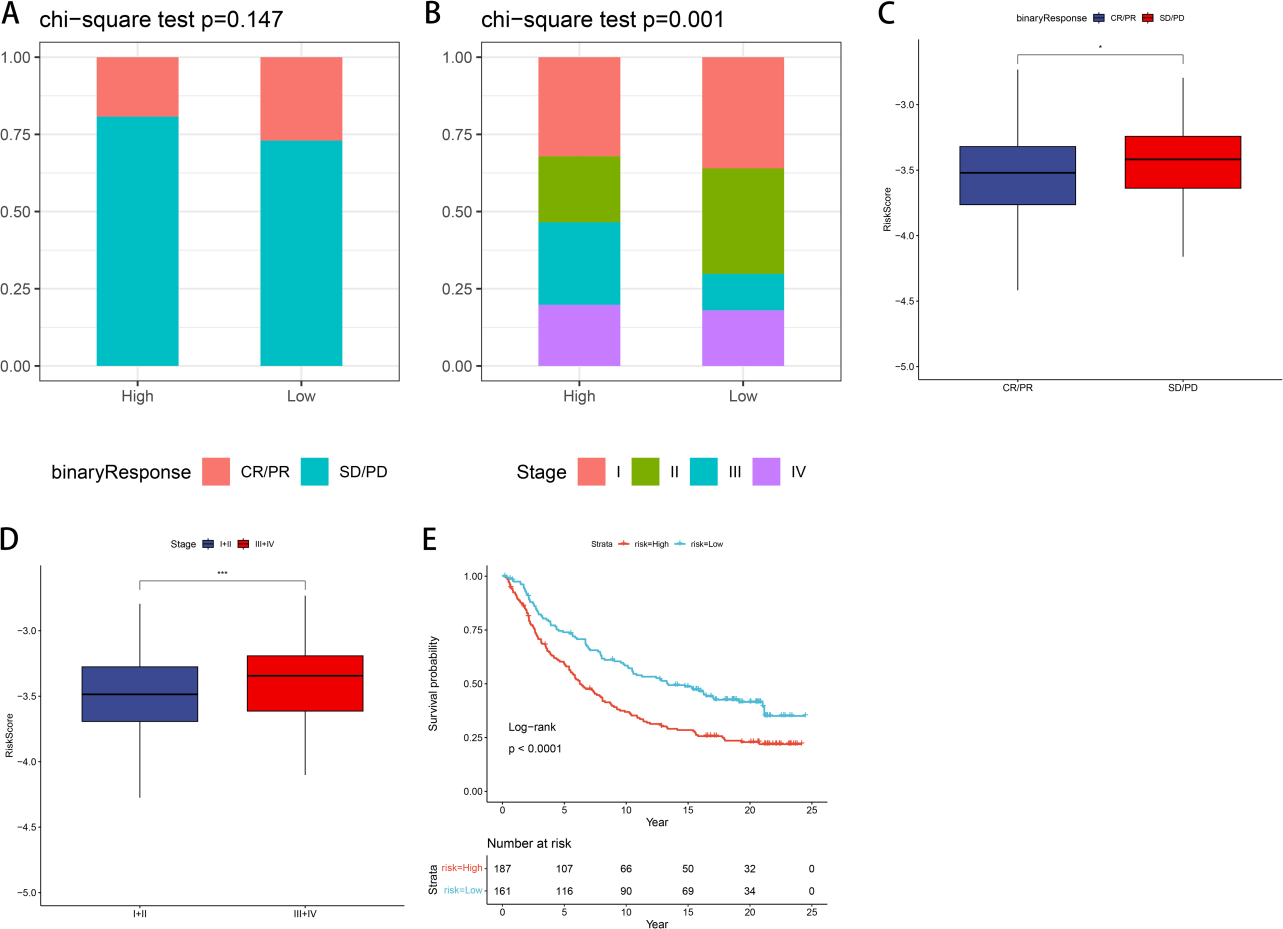
Validation of the risk model using the IMvigor210 immunotherapy cohort. **(A)** No significant difference in treatment response (CR/PR vs. SD/PD) between risk groups. **(B)** Early-stage patients (stage I+II) are more common in the low-risk group (*P*=0.001). **(C, D)** Risk score is higher in SD/PD and stage III+IV groups (*P*<0.05, *P*<0.001). **(E)** K-M analysis shows superior prognosis for low-risk patients (*P*<0.0001). CR, complete response; PR, partial response; SD, stable disease; PD, progressive disease. *p<0.05, ***p<0.001.

We validated our prognostic model using the IMvigor210 dataset, investigating immunotherapy response efficacy in patients categorized as complete or partial response (CR/PR) versus stable disease or progressive disease (SD/PD) based on risk within GNG7 high- and low-risk groups. There was no significant difference noted in the evaluation of treatment response (**Figure 9A**). However, when stratified by cancer stage, a significant association was noted, with a higher proportion of early-stage patients identified in the low-risk group (*P*=0.001, **Figure 9B**). Furthermore, concerning risk score, the risk score of the SD/PD group was significantly higher compared to that of the CR/PR group (*P*<0.05, **Figure 9C**). Comparison of risk scores between early-stage (I+II) and late-stage (III+IV) cancers revealed stage-dependent differences, with higher risk scores observed in later stages (*P*<0.001, **Figure 9D**). In addition, the K-M curve shows that compared to the high-risk group, patients in the low-risk group have significantly better prognosis (*P*<0.0001, **Figure 9E**).

### Construction and validation of predictive nomograms

**Figure 10:**
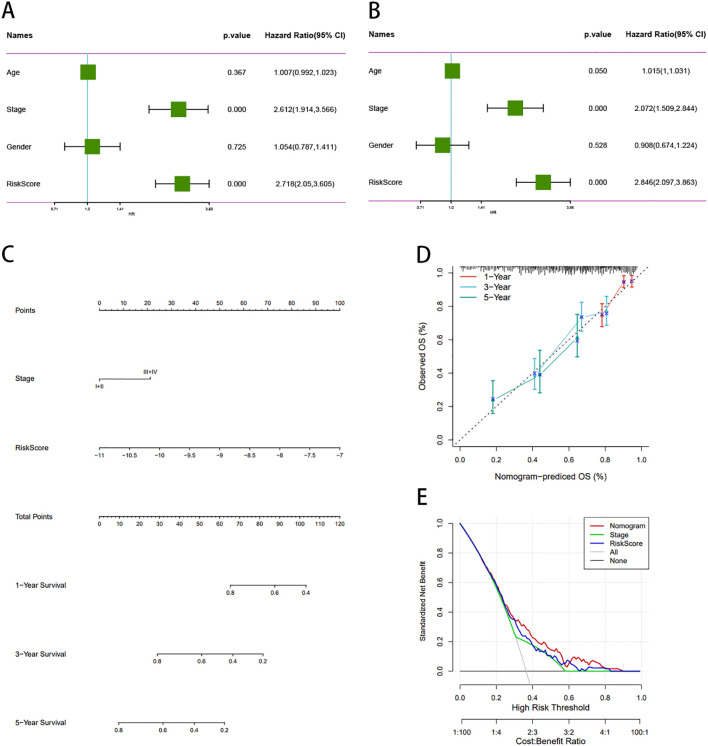
Construction and validation of a prognostic nomogram for LUAD patients. **(A, B)** Multivariate Cox regression confirms stage and risk score as independent prognostic factors. **(C)** Nomogram predicts 1-, 3-, and 5-year OS based on clinical and molecular features. **(D)** Calibration plots show high concordance between predicted and observed OS. **(E)** DCA curve demonstrates superior clinical utility of the nomogram. DCA, decision curve analysis.

We assessed this model using both univariate and multivariable Cox regression analyses on other clinical variables, encompassing age, stage, sex, and risk score, to determine whether it remained independent of other clinical prognostic factors that might influence patient outcomes. Stage and risk score were identified as independent predictors of OS (**Figure 10A-B**). Nomograms constructed from these independent prognostic biomarkers can be used to quantify individual 1-, 3-, and 5-year survival (**Figure 10C**). The calibration curve demonstrated high consistency between the expected OS at 1, 3, and 5 years and the observed OS. (**Figure 10D**). In addition, we constructed a norm chart to predict patient prognosis, and the results showed that it was basically consistent with clinical prediction. Decision curve analysis (DCA) compared the net benefits of different risk thresholds and proved that normgram had the best prediction effect (**Figure 10E**).

### GNG7 Expression and its role in cell proliferation and apoptosis in LUAD

**Figure 11:**
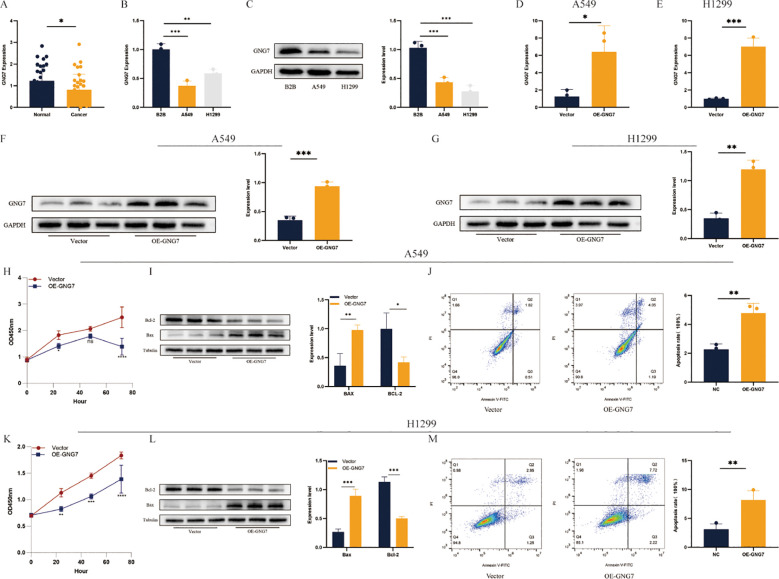
GNG7 inhibits LUAD cell proliferation and induces apoptosis. **(A, B)** qPCR shows GNG7 is downregulated in LUAD tissues and cell lines (*P*<0.05, *P*<0.01). **(C)** Western blot confirms decreased GNG7 protein in A549/H1299 cells (*P*<0.001). **(D–G)** GNG7 overexpression increases mRNA and protein levels (*P*<0.05). **(H, K)** CCK-8 assays show reduced proliferation with GNG7 overexpression. **(I, L)** WB reveals increased BAX and decreased BCL-2 levels (*P*<0.05). **(J, M)** Flow cytometry confirms increased apoptosis in GNG7-overexpressing cells (*P*<0.01). *p<0.05, **p<0.01, ***p<0.001.

qPCR analysis of 34 LUAD samples revealed a significant decrease in GNG7 expression in tumor tissues compared to adjacent normal tissues (**Figure 11A**, P < 0.05). Similarly, qPCR results showed a marked reduction in GNG7 expression in A549 and H1299 cells relative to B2B cells (**Figure 11B**, *P*<0.01). WB further confirmed these findings, showing lower GNG7 protein levels in A549 and H1299 cells compared to B2B cells (**Figure 11C**, *P*<0.001). GNG7 overexpression in A549 and H1299 cells led to a significant increase in both mRNA and protein levels (**Figures 11D-G**, *P*<0.05), and CCK-8 assays demonstrated a notable decrease in cell proliferation (**Figures 11H, K**). WB analysis showed increased expression of the pro-apoptotic protein BAX and decreased expression of the anti-apoptotic protein BCL-2 following GNG7 overexpression (**Figures 11I, L**, *P*<0.05). Additionally, flow cytometry analysis confirmed a significant increase in apoptosis in GNG7-overexpressing cells (**Figures 11J, M**, *P*<0.01).

### GNG7 overexpression inhibits cell migration and invasion in A549 and H1299 cells

**Figure 12:**
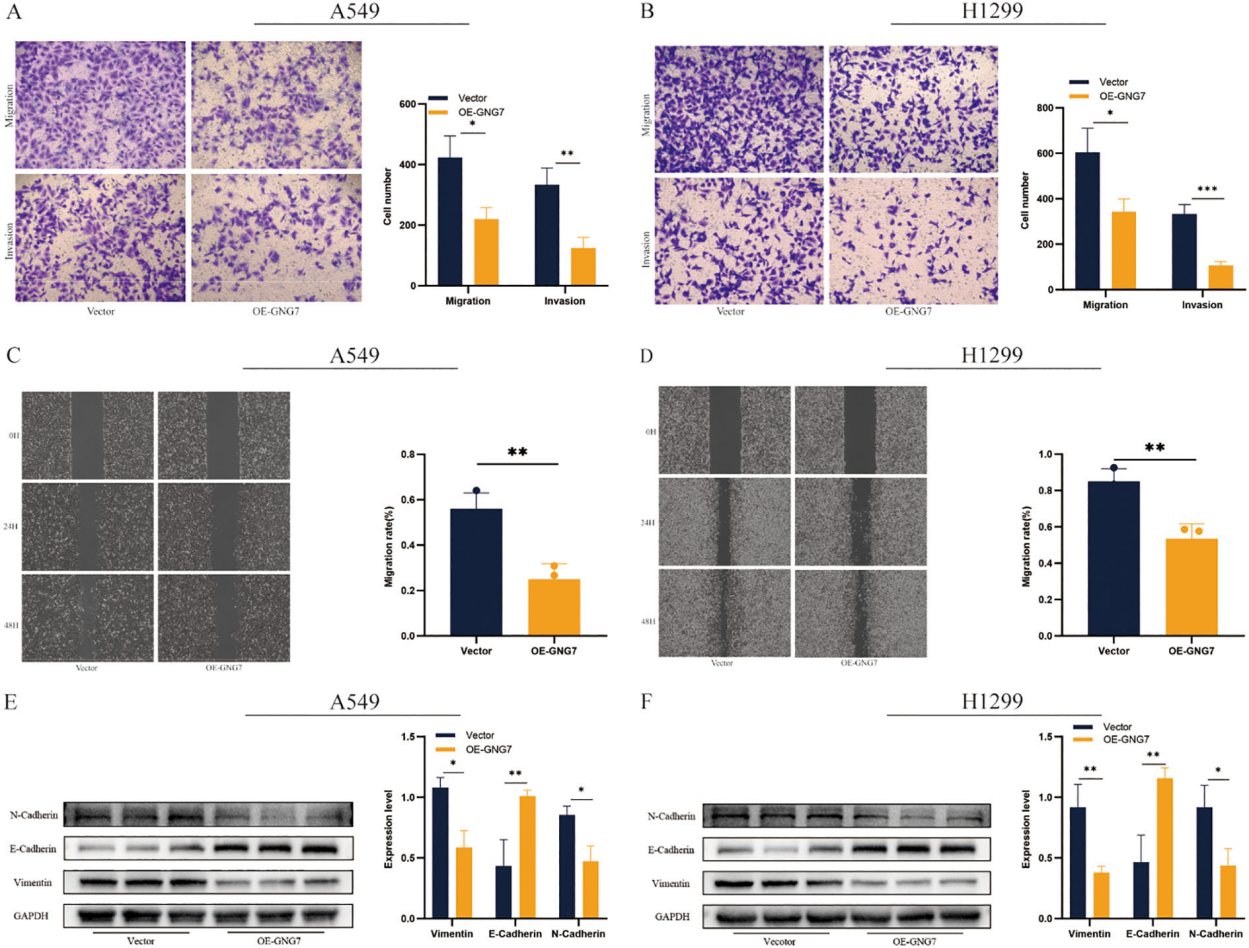
GNG7 suppresses LUAD cell migration and invasion. **(A, B)** Transwell assays show reduced invasion/migration in GNG7-overexpressing cells (*P*<0.05). **(C, D)** Scratch assays confirm impaired wound healing ability (*P*<0.01). **(E, F)** Western blot shows increased E-Cadherin and reduced N-Cadherin and Vimentin in GNG7-overexpressing cells (*P*<0.05). *p<0.05, **p<0.01, ***p<0.001.

Overexpression of GNG7 significantly reduced the invasion and migration abilities of A549 and H1299 cells, as shown by invasion and migration assays (**Figures 12A, B**, *P*<0.05). The scratch assay further confirmed this result, demonstrating that GNG7 overexpression significantly slowed the wound healing ability of these cells (**Figures 12C, D**, *P*<0.01). Western blot analysis revealed that GNG7 overexpression upregulated the expression of E-Cadherin, while downregulating the expression of N-Cadherin and Vimentin (**Figures 12E, F**, *P*<0.05).

## Discussion

Lung cancer continues to be a prominent contributor to cancer-related mortality worldwide, with LUAD in the spotlight due to its complex molecular mechanisms and diverse treatment responses (19, 20). Identifying and understanding the molecular mechanisms of this disease’s progression is an urgent task in current research. The morphological differences and molecular heterogeneity of tumors are crucial for the diagnosis and treatment of LUAD, highlighting the necessity of discovering new, convenient, non-invasive immunobiological markers to enhance the detection and treatment outcomes of LUAD (21–23). The efficacy and safety of immunotherapy have been confirmed through numerous clinical and experimental studies, highlighting the promising potential of immunotherapy in managing LUAD.

GNG7 is a crucial factor in cellular signal transduction, involved in various biological processes. Research has shown that GNG7 can induce apoptosis and halt the cell cycle by diminishing the assembly of actin filaments (24–26). GNG7 inhibits the development of LUAD by targeting the E2F1 and Hedgehog signaling pathways (27), and enhances immune responses by increasing B cell infiltration and MHCII antigen processing, thus modulating the TME in LUAD (28). Our study observed that low expression of GNG7 is positively correlated with poorer OS, suggesting that GNG7 exerts a protective role by inhibiting tumor growth and facilitating apoptosis of tumor cells. Tumor cells avoid detection and elimination by the host immune system through various mechanisms, increasing the risk of tumor occurrence when immune function is impaired or suppressed (29, 30). Tumor cells may alter the expression of surface antigens, secrete immunosuppressive factors, induce the aggregation of immunosuppressive cells such as Tregs, and inhibit T cell activity through immune checkpoint pathways like the PD-1/PD-L1 axis (31, 32).

Given GNG7’s role in intracellular signaling and actin cytoskeleton regulation, it is plausible that it affects tumor immune evasion by modulating cytokine secretion and immune cell trafficking, thereby altering immune recognition and response. Specifically, GNG7 can modulate immune cell recruitment at the molecular level by influencing the expression of chemokines and antigen-presenting molecules such as MHC-II. These molecules play a crucial role in regulating immune cell infiltration patterns within the tumor microenvironment (TME). Specifically, GNG7 may enhance the recruitment and activation of CD8+ T cells and NK cells by increasing chemokine signaling, which is vital for antitumor immunity. Furthermore, GNG7’s ability to modulate antigen presentation could potentially improve the visibility of tumor cells to immune cells, promoting more robust immune responses against the tumor.

Recently, the immune evasion mechanisms in tumors, particularly those related to immune cell death, have garnered increasing attention. For instance, cuproptosis, a copper-dependent cell death mechanism, has been shown to play a critical role in regulating the tumor microenvironment in certain cancers (33, 34). Although its direct role in LUAD remains unexplored, similar immune evasion mechanisms may exist. GNG7, a key regulator of immune responses, could influence immune cell recruitment, cytokine secretion, and antigen presentation, thereby modulating tumor immune evasion. Future studies should explore the relationship between GNG7 and immune-related cell death mechanisms like cuproptosis to identify new therapeutic targets for LUAD immunotherapy.

Moreover, GNG7 may also be involved in the modulation of immune checkpoint pathways, such as the PD-1/PD-L1 axis (35, 36), indirectly enhancing T cell activity by reducing tumor-induced immune suppression (37, 38). This suggests that GNG7 might enhance the efficacy of immune checkpoint inhibitors by creating a more immunoreactive tumor environment. These findings imply that GNG7 could play a key role in modulating immune checkpoints, which are crucial in tumor immune escape mechanisms.

Pathway enrichment analysis further revealed that high GNG7 expression was significantly associated with upregulation of several immune-related pathways. These findings suggest that GNG7 may enhance immune surveillance by promoting antigen visibility, activating innate cytotoxic responses, and modulating cytokine signaling in the tumor microenvironment. However, it is important to consider that TCGA data, while comprehensive, may introduce certain biases. Previous studies have highlighted limitations in TCGA datasets, such as the heterogeneity of sample origins and the potential for batch effects due to the inclusion of samples from multiple institutions (39). These factors can introduce variability in gene expression profiles and immune-related pathway analyses, which could influence the observed relationships between GNG7 and immune responses. Additionally, the representation of diverse ethnic groups and the absence of longitudinal data in TCGA may further limit the generalizability of findings across different populations and clinical settings.

In support of these hypotheses, pathway enrichment analysis further revealed distinct differences between high and low GNG7 expression groups. High GNG7 expression was significantly associated with upregulation of several immune-related pathways, including antigen processing and presentation, natural killer cell-mediated cytotoxicity, and cytokine–cytokine receptor interaction. These findings suggest that GNG7 may enhance immune surveillance by promoting antigen visibility, activating innate cytotoxic responses, and modulating cytokine signaling in the tumor microenvironment.

In contrast, low GNG7 expression was enriched in oncogenic and metabolic pathways such as cell cycle, p53 signaling pathway, and glycolysis/gluconeogenesis. This pattern indicates that reduced GNG7 expression may contribute to uncontrolled cell proliferation, altered tumor suppressor responses, and metabolic reprogramming—hallmarks of cancer progression and immune evasion. These results reinforce the potential of GNG7 as a key modulator of both tumor growth and immune activity in LUAD.

*In vitro* experiments have further confirmed these effects, revealing that overexpression of GNG7 significantly inhibits cell proliferation, invasion, and migration, while promoting apoptosis in LUAD cells. These findings highlight the potential of GNG7 as a tumor suppressor gene in LUAD, contributing not only to immune modulation but also to the direct suppression of tumor growth and metastasis. By reducing the proliferative capacity and metastatic potential of LUAD cells, GNG7 could serve as a critical factor in controlling tumor progression and improving patient prognosis.

The Tumor Immune Microenvironment (TIME) plays a pivotal role in cancer progression, encompassing immune cells, cytokines, blood vessels, and the extracellular matrix around tumor cells. This complex network significantly influences tumor growth, spread, and response to therapy (40). Among the key players in the adaptive immune system, T cells stand out for their ability to specifically bind tumor antigens via surface receptors, thereby initiating an immune response. Their high specificity and potent antitumor activity make them crucial in cancer immunotherapy (41, 42). Tumor-infiltrating lymphocytes (TILs), particularly cytotoxic T lymphocytes (CTLs), play a crucial role in shaping the TIME by eliminating tumor cells. This process promotes the release of antigens and damage-associated molecular patterns (DAMPs) (43). Nevertheless, the association between CD8+ T cells and LUAD prognosis remains contentious due to variations in distribution sites, pathological classifications, or cell quantification methods. However, multiple studies have consistently linked elevated levels of CTLs in both the tumor and stroma with positive outcomes in lung cancer (44, 45).

Accumulation or activation of Treg cells in lung cancer patients has been demonstrated. These cells maintain immune tolerance to self-antigens during carcinogenesis, facilitating tumor evasion and contributing to the suppression of antitumor immunity (46, 47). TILs, comprising cells involved in the innate immune response such as effector T cells and NK cells, have been demonstrated to positively correlate with lung cancer prognosis. This positioning lung cancer as an ideal candidate for TIL adoptive cell therapy (48). This therapy involves extracting and expanding tumor-specific lymphocyte colonies *in vitro (* 49). A phase I open label trial investigated the safety and initial efficacy of TIL treatment combined with nivolumab in the treatment of advanced NSCLC, and compared it with nivolumab monotherapy. The results indicate that the majority of patients have achieved confirmed clinical remission (50).

Tumor-associated macrophages (TAMs) play a crucial role in the TME by influencing cancer progression. TAMs exhibit a plasticity that allows them to adopt different phenotypes—M1 or M2—depending on the environmental cues present within the TME (51, 52). M1 macrophages, typically associated with pro-inflammatory responses, actively participate in the destruction of tumor cells, antigen presentation, and immune surveillance. In contrast, M2 macrophages, which dominate the TME in many cancers, suppress immune responses, favor tumor survival, and promote metastasis (53). GNG7 may play a pivotal role in skewing macrophage polarization, potentially encouraging a shift toward a more immune-activating M1 phenotype that enhances antitumor immunity while reducing immune suppression.

Moreover, GNG7’s influence on immune cell recruitment extends beyond macrophages and T cells. The tumor microenvironment can modulate the infiltration of various immune cell types, including regulatory T cells (Tregs) and mast cells, which can either support or hinder immune responses. In this context, GNG7’s ability to modulate the immune landscape could enhance tumor-specific immune responses, making it a valuable target for immunotherapies, including CAR-T cell therapies (54).

As for newer therapeutic approaches such as CAR-T cell therapy, which involves modifying T cells to better target tumor cells (37), GNG7’s role in enhancing immune cell recruitment and activation may synergize with CAR-T cell therapy. GNG7 could potentially improve the efficacy of CAR-T therapies by fostering a more favorable immune environment that supports T cell activity, reduces immune suppression, and enhances the homing and infiltration of T cells into tumors. This combined approach could significantly improve therapeutic outcomes for patients with lung adenocarcinoma (LUAD), particularly those who are resistant to traditional treatments.

Our study found that high immune scores of NK cells, CD8+ T cells, M2 macrophages, and CTLs were significantly enriched in the GNG7 low-risk group, while high immune scores of Treg cells were negatively associated with this group (p < 0.05, Wilcoxon rank-sum test). These findings suggest that GNG7 may shape a more immunoreactive tumor microenvironment (TME) by enhancing the recruitment or activation of antitumor immune cells while suppressing immunosuppressive populations. Similar analyses using TCGA data have also explored immune cell infiltration patterns in lung adenocarcinoma (LUAD) (55). For example, previous studies have demonstrated associations between immune-related gene signatures and patient prognosis, supporting the relevance of immune cell activity in LUAD. However, variations in the immune profile across TCGA cohorts could be attributed to the heterogeneity of data sources, highlighting the importance of context when interpreting immune infiltration data from TCGA (56).

Additionally, GNG7 might influence macrophage polarization and T cell activation, further contributing to a favorable immune landscape. This pattern underscores the potential of GNG7 as an immunological marker for risk stratification in LUAD, and possibly as a predictive biomarker for immunotherapy efficacy, especially checkpoint blockade strategies.

Our study uncovered a positive correlation between the expression of mast cells and the expression level of GNG7, particularly in the GNG7 low-risk group. While mast cells are traditionally associated with allergic diseases, mounting evidence in recent years has indicated their involvement in cancer, such as LUAD (57–59). Mast cells are involved in regulating disease processes such as inflammation and tissue remodeling, and are associated with innate and adaptive respiratory pathogen immune responses (60). Considering the critical role of the immune microenvironment in tumor progression, mast cells, as essential components of the immune system, undoubtedly act as key regulators in maintaining tissue homeostasis. Whether GNG7 directly modulates mast cell activity remains unknown, but this connection warrants further investigation in the context of LUAD. Thus, we advocate for further exploration of the role of mast cells in reshaping the TME.

In recent years, with the continuous advancement of lung cancer treatment strategies, genes such as HER2, KRAS, and NTRK have emerged as significant targets for targeted therapy in NSCLC. Despite some progress, resistance to targeted drugs in advanced NSCLC patients continues to pose a significant challenge (61). Fortunately, immunotherapy checkpoint inhibitors targeting PD-1/PD-L1 and CTLA-4 have opened new avenues for NSCLC treatment. It’s worth noting that the expression of PD-1 and its ligand PD-L1 is upregulated upon T cell activation (62), and their interaction inhibits T cell activity, thereby hindering the attack of cytotoxic T lymphocytes (CTL) on tumor cells, leading to CTL dysfunction and immune escape (63). Additionally, the interaction between PD-1 and PD-L1 can suppress T cell proliferation and the production of crucial cytokines such as IL-2 and IFN-γ. Moreover, it can influence B cell functions, consequently weakening the overall immune response (64). Elevated expression of PD-L1 in cancer tissues is frequently linked to a high degree of T cell infiltration, which typically signifies T cell exhaustion and a diminished anti-tumor function. This phenomenon allows the tumor to evade surveillance and elimination by the immune system (65). Overall, immune checkpoints such as PD-1/PD-L1 and CTLA-4 tend to attenuate the regulatory effects of immune cells on tumor immunity, ultimately resulting in tumor cell escape. At present, tissue-based biomarkers including PD-L1 expression level, tumor mutation burden (TMB), microsatellite instability (MSI), mismatch repair (MMR) gene defect and TIL have been proved to be reliable in predicting the effectiveness of immunotherapy for NSCLC (66, 67). With the application of immune checkpoint inhibitors in the treatment of adenocarcinoma, exploring the interaction between GNG7 and these treatment strategies has become particularly important. If GNG7 indeed facilitates tumor-specific immune responses, its expression could serve not only as a predictive biomarker for the efficacy of immunotherapy, but also as a potential therapeutic target to reverse resistance to immune checkpoint inhibitors (ICIs). High GNG7 expression may correlate with greater infiltration of effector immune cells and reduced presence of Tregs, both of which are known indicators of favorable immunotherapeutic responses.

However, it is important to note that the use of external datasets, such as the IMvigor210 cohort, primarily focusing on urothelial carcinoma (UC) for immunotherapy validation, may have potential limitations when applied to LUAD. The immune responses and tumor microenvironment characteristics in UC could differ significantly from those in LUAD. For example, the immune cell infiltration patterns, immune checkpoint expression, and immune evasion mechanisms may vary, limiting the direct applicability of findings from the IMvigor210 cohort to LUAD. Therefore, while the IMvigor210 cohort offers valuable insights, caution should be taken when extrapolating these results to LUAD, and LUAD-specific validation studies are necessary for more accurate conclusions regarding immunotherapy efficacy.

Future clinical validation could determine whether GNG7 expression levels correlate with treatment outcomes in patients receiving ICIs, such as anti–PD-1 or anti–CTLA-4 agents. Stratifying patients by GNG7 expression might also help optimize immunotherapy decisions and improve personalized treatment strategies (68).

Future research should aim to deepen our understanding of how GNG7 regulates immune responses at the molecular level, including its role in immune escape in lung adenocarcinoma. Establishing different cell models will be crucial for this purpose, allowing for more precise exploration of GNG7’s function in diverse tumor microenvironments (69). Additionally, exploring interactions between GNG7 and other immune regulatory factors could reveal new immunotherapeutic targets. Ultimately, validating the correlation between GNG7 expression levels and immune therapy responses through clinical studies will provide personalized and combinational treatment options for lung adenocarcinoma patients. Furthermore, enhancing the safety of immunotherapy and reducing side effects to ensure the quality of life for patients will be another key direction of research.

Indeed, recognizing the limitations associated with utilizing open datasets and the necessity of supplementing patient samples and fundamental assays to validate the significance and value of GNG7 in LUAD is crucial. Further research should include more comprehensive clinical trials to study the influence of GNG7 on the recruitment of immune cells, infiltration, and immune therapy responses at the cellular and molecular levels. These studies will enhance our understanding of GNG7’s function in LUAD and its potential impact on future treatments and diagnostics. Insights gained from the research lay the groundwork for further advancements in the LUAD field and provide valuable directions for future studies.

## Conclusion

Our results show that GNG7 is downregulated in various cancers, including LUAD, and its expression is associated with patient prognosis. Higher GNG7 expression correlates with better survival in some cancers, while lower expression is linked to poorer outcomes, particularly in LUAD. Overexpression of GNG7 inhibits cell proliferation, migration, and invasion, while promoting apoptosis. It also affects immune cell infiltration, suggesting that GNG7 could be a potential prognostic biomarker and therapeutic target, especially in immunotherapy for LUAD.

## Data Availability

Requests to access the datasets should be directed to https://www.cancer.gov/ccg/research/genome-sequencing/tcga.
